# The Roles of Glucagon-Like Peptide 1 (GLP-1) Receptor Agonists and Sodium-Glucose Cotransporter 2 (SGLT-2) Inhibitors in Decreasing the Occurrence of Adverse Cardiorenal Events in Patients With Type 2 Diabetes

**DOI:** 10.7759/cureus.33484

**Published:** 2023-01-07

**Authors:** Dalia Shami, John M Sousou, Einas Batarseh, Laith Alazrai

**Affiliations:** 1 General Practice, AlMahabba Hospital, Madaba, JOR; 2 Internal Medicine, Lake Erie College of Osteopathic Medicine, Jacksonville, USA; 3 Internal Medicine, University of Buffalo, Buffalo, USA; 4 Internal Medicine, Jordanian Royal Medical Services, Amman, JOR

**Keywords:** cardiovascular disease risk, hyperglycemia management, cardio-renal cascade, sodium-glucose cotransporter-2 (sglt-2) inhibitors, glp-1 receptor agonists, type-2 diabetes mellitus

## Abstract

Diabetes mellitus is a metabolic disorder characterized by increased serum glucose due to errors in insulin production or response. The prevalence of diabetes mellitus has continued to rise globally over the years, with roughly 7079 persons per 100,000 expected to be impacted by 2030. A vast number of patients with diabetes mellitus experience unfavorable side effects such as weight gain, hypoglycemia, and hepatorenal toxicity from the several diabetic medications available. These adverse effects may result in life-threatening consequences with a high likelihood of occurrence; therefore, ongoing efforts continue to develop medications with improved tolerability and better glycemic control. Glucagon-like peptide-1 receptor agonists (GLP-1RA) and sodium-glucose cotransporter 2 inhibitors (SGLT-2i) are examples of new innovative targeted therapies to manage diabetes mellitus and potentially improve cardiorenal conditions. This review article details the specific mechanisms of action, potential side effects, and cardiorenal benefits of GLP-1RA and SGLT-2i therapies to fully understand their roles in combating type 2 diabetes mellitus (T2D).

## Introduction and background

Diabetes mellitus is a disorder characterized by decreased metabolic function as a result of defective insulin production and response, leading to serious health complications [1]. Type 2 diabetes mellitus (T2D) has evolved over the years into a global health concern impacting millions worldwide. In 2017, it was estimated that about 462 million people suffer from T2D globally, which accounts for around 6.28% of the world’s population. In addition, research suggests that the global prevalence of T2D will continue to rise, with roughly 7079 persons per 100,000 affected by 2030 [2]. These numbers are alarming, not only due to the large number of people suffering from T2D but because it can also lead to other major conditions, such as chronic kidney disease (CKD) and cardiovascular disease (CVD). T2D has played a substantial role in the significant increase of CKD cases globally over the past several decades [3]. CVD has also been a major concern for patients with T2D, as they have a considerably increased risk of experiencing major cardiovascular conditions, such as coronary artery disease, heart attack, and stroke [4]. Diabetes-associated risks must be tackled and minimized to preserve the health of those most vulnerable to them.

Despite the healthcare industry's best efforts, many people with diabetes mellitus have encountered unfavorable side effects such as weight gain, hypoglycemia, and hepatorenal toxicity from medications. These adverse effects may result in life-threatening consequences with a high likelihood of occurrence; therefore, ongoing efforts continue to develop medications with improved tolerability and better glycemic control [5]. Glucagon-like peptide-1 receptor agonists (GLP-1RA) and sodium-glucose cotransporter 2 inhibitors (SGLT-2i) are examples of new innovative targeted therapies to manage diabetes mellitus and potentially improve cardiorenal conditions. Studies have shown that SGLT-2i plays a pivotal role in glucose homeostasis in the kidneys. Thus, pharmacological inhibition of SGLT-2i holds great potential for treating T2D, whereas GLP-1RA was studied to determine its efficacy in preventing cardiovascular conditions in T2D patients [5]. Initially, the use of GLP-1RA for the treatment of diabetes raised some safety concerns for patients with medullary thyroid carcinoma, pancreatitis, and pancreatic cancer. Cardiovascular trials, however, showed minimal safety signals when using GLP-1RA, which even reduced the rates of stroke, myocardial infarction, and cardiovascular death [1]. Other cardiovascular trials run on T2D patients who have had standard medical therapy have also shown a reduction in Major Adverse Cardiovascular Events (MACE) rates when using GLP-1RA or SGLT-2i. While specific medications under both classes of drugs have shown benefits in preventing the occurrence of cardiovascular events, the difference lies in the type of MACE they are preventing. SGLT-2i are preferred in diabetics with heart failure (HF), whereas GLP-1RA is addressed for atherosclerotic CVD. Ultimately, both classes have shown significant results in reducing the risk of cardiorenal disease [4].

## Review

GLP-1 receptor agonists

Pharmacological Action

One of the glucose-reducing therapies being studied to help treat T2D is GLP-1RA. This medication primarily increases the levels of incretin, a hormone that aids in regulating postprandial insulin secretion. GLP-1RA not only help pancreatic beta cells release glucose-dependent insulin but also makes pancreatic alpha cells more susceptible to glucose, causing decreased secretion of glucagon and thus reducing the amount of endogenous glucose produced. By decreasing glucagon production, the amount of insulin the body needs will decrease, ultimately improving control of glucose levels. Certain studies have shown that GLP-1RA may also act on peripheral glucose found in the central nervous system (CNS). In animals, for instance, increasing energy expenditure by stimulating GLP-1 receptors has shown a reduction in food intake due to brown adipose tissue thermogenesis and white adipose tissue browning. Furthermore, GLP-1RA can also reduce the body’s overall glucose absorption and postprandial glucose by delaying gastric emptying. This occurs when the vagus nerve is stimulated by GLP-1 receptors found in the CNS and vagal efferent fibers in the brainstem. The vagus nerve will then inhibit gastric emptying and gastric acid secretion, which subsequently results in a delay of gastric emptying and reduction of glucose absorption [6].

While the long-term use of GLP-1RA did not show significant weight loss effects on animals, studies have shown the opposite when these drugs were administered to humans. Prolonged use of GLP-1RA has been shown to stimulate weight loss. By targeting GLP-1 receptors in hypothalamic satiety centers of the brain, GLP-1RA can be used to control appetite and thus help in weight loss due to the brain and stomach both acting on central and peripheral receptors. In addition and similar to animal trials, GLP-1RA results in decreased gastric emptying to the small intestine and gives the feeling of being full [7].

Adverse Drug Reactions

When taking GLP-1RA, it is instrumental to note the potential side effects that might arise. The most common side effect is gastrointestinal discomforts, such as nausea, bloating, and abdominal pain, which may result in patients discontinuing their medication. GLP-1RA can potentially cause more serious issues, such as pancreatitis and pancreatic cancer [4]. Furthermore, stimulation of GLP-1 receptors located on myocytes in the SA (sinoatrial) node of the human heart can lead to transient tachycardia, likely due to β-adrenergic stimulation and subsequent vasodilation [7].

Roles and Benefits

The different types of medications within the GLP-1RA class have shown various effects on CVD and weight loss. Although its true mechanism of action is somewhat vague, several studies have concluded that the impact of GLP-1RA is a result of anti-atherogenic mechanisms through its effect on body weight and lipid profiles. These medications induce significant weight loss due to their role in regulating glucose by decreasing gastric emptying, stimulating glucose-dependent insulin secretion, suppressing glucagon secretion, and decreasing appetite. GLP-1RA have shown success in metabolic syndromes due to their benefits; semaglutide and liraglutide have received FDA approval for weight loss even without the presence of diabetes [8]. Furthermore, GLP-1RA have a minimal incidence of causing hypoglycemia. In addition, the GLP-1 receptors found in the heart and endothelial tissue contribute to anti-inflammatory pathways and have direct effects on the myocardial and/or vascular endothelium [7]. While studies are still being undertaken to evaluate the full effect of GLP-1RA on cardiovascular disease, certain trials have shown positive results with daily liraglutide or weekly semaglutide administration (Table 1). Another trial shows that although weekly exenatide ER did not significantly reduce myocardial infarction, stroke, or hospitalization rate for heart failure, it was as safe as the placebo effect [7]. Overall, the use of GLP-1RA was associated with a 14% decrease in overall mortality and a 12% reduction in cardiovascular mortality [9]. Some cardiovascular trials have shown that the use of GLP-1RA can reduce the occurrence of stroke by around 13%, excluding protection from fatal strokes. In addition, studies have also shown the renal impact of using GLP-1RA treatment on patients with T2D. In one study conducted on animals, GLP-1RA were shown to reduce oxidation, inflammation, albuminuria, and glomerular sclerosis, all while protecting the vascular endothelium [10]. The cardiorenal benefits of GLP-1RA are summarized in Table 1 below.

**Table 1:** Comparisons among specific GLP-1RA GLP-1RA, Glucagon-like peptide-1 receptor agonists; GLP-1, Glucagon-like peptide-1; eGFR, estimated glomerular filtration rate; UACR, urine albumin to creatinine ratio; CKD, chronic kidney disease; MACE, major adverse cardiovascular events

GLP-1 Receptor Agonist	Weight Loss	Blood Pressure	Renal Effect	Cardiac Effect	Stroke Protection
Exenatide ER (EXSCEL) [7,11,12]	Moderate weight loss	Significantly reduces systolic blood pressure (~2 mmHg)	N/A	Reduces the risk of cardiovascular events	Helps reduce the risk of total stroke by 13% (excluding fatal strokes)
Liraglutide (LEADER) [11,13]	Reduces weight loss significantly	Reduces systolic blood pressure with no effect on diastolic blood pressure (but the difference between it and placebo was not significant after 1 year of administration)	Reduces new onset macroalbuminuria and chronic kidney disease progression	Reduces the risk of cardiovascular events with daily liraglutide	Helps reduce the risk of total stroke by 13% (excluding fatal strokes)
Lixisenatide (ELIXA) [11,14,15]	Moderate weight loss	Reduced any decrease in systolic blood pressure	-Reduces the development of UACR -Reduces the risk of new onset microalbuminuria -No effect on GFR decline	Safe but did not show improvements in cardiovascular morbidity and mortality	Helps reduce the risk of total stroke by 13% (excluding fatal strokes)
Dulaglutide (AWARD-7) (REWIND) [11,12,15]	Its large size plays a role in making it less effective in weight loss and satiety	No clear results, but some studies showed no major impact	-Helps prevent the decline of eGFR -Helps reduce UACR -Helps preserve renal function (primarily in patients with macroalbuminuria and CKD stage 3b and 4)	Reduces the risk of cardiovascular events	- Reduces the occurrence of ischemic stroke (but not the severity) - No effects were seen on hemorrhagic stroke
Semaglutide (PIONEER 6) (SUSTAIN) [7,11,12,15]	Most effective in weight loss	Significantly reduces systolic blood pressure (~2 mmHg)	Reduces new onset macroalbuminuria and chronic kidney disease progression	-Weekly semaglutide helps reduce the risk of cardiovascular events -Helps reduce cardiovascular death -Most effective in preventing MACE	- Helps reduce the risk of total stroke by 13% (excluding fatal strokes) - Helps reduce strokes

SGLT-2 inhibitors

Pharmacological Action

Similar to GLP-1RA, SGLT-2i are being used to decrease blood glucose levels in T2D patients. Although both medications have a similar role, they have different mechanisms of action. When used as a treatment to reduce glucose levels in the body, SGLT-2i cause glycosuria by inhibiting renal tubular glucose reabsorption; this causes glucose and sodium excretion through the urine [16]. Several clinical trials have shown significant benefits in the use of SGLT-2i on T2D patients with cardiovascular and/or renal disease [17]. One study demonstrated a significant reduction (38%) in the occurrence of cardiovascular death and stroke in T2D patients taking empagliflozin [18]. Additionally, hospitalization due to heart failure in these patients was reduced by 35%, along with all-cause mortality being reduced by 32%.

T2D patients with severe renal impairment who take SGLT-2i will likely need dose monitoring since the pharmacodynamic response tends to decline with the use of SGLT-2i. Blood pressure and serum lipid profiles may also be affected by SGLT-2i, which entails both a positive and negative outcome. SGLT-2i may increase high-density lipoprotein (HDL) cholesterol and decrease triglycerides, which is considered to be beneficial; however, they have also been shown to increase low-density lipoprotein (LDL) cholesterol [19]. SGLT-2i also significantly reduces serum uric acid levels in the body by interacting with GLUT-9 (SLC2A9), a facilitated glucose transporter that secretes uric acid into the urine and thus decreases serum uric acid in the body [20]. This ultimately results in a decreased risk of cardiovascular disease, nephrolithiasis, and gout.

Adverse Drug Reactions

Similar to most medications, SGLT-2i has several side effects that must be taken into consideration before being prescribed. The most common side effects of SGLT-2i include female genital mycotic infections, urinary tract infections, and increased urination [19]. However, other studies also suggest that there is no increased risk of urinary tract infections (UTIs) associated with the use of SGLT-2i, except for a prior history of UTIs. With T2D patients already at a higher risk of developing diuresis and fungal genital infections, using SGLT-2i may result in polyuria, especially shortly after initial administration [11].

Less common side effects include diabetic ketoacidosis, and breast and bladder cancer when dapagliflozin was used [19]. In addition, there were some rising concerns about Fournier’s gangrene, but no increased risk was highlighted in any of the significant trials [10]. Euglycemic DKA (diabetic ketoacidosis) is also another rare but serious side effect of using SGLT-2i. Moreover, the role of SGLT-2i on the kidneys may result in renal reabsorption of ketones and increased circulating levels, thus placing the patient at increased risk of acidemia by ketogenesis.

Roles and Benefits

SGLT-2i stimulates mild diuresis and aids in secreting glucose into the urine, which helps decrease blood pressure and stimulate weight loss, respectively [21]. While SGLT-2i can be used individually to reduce weight, its effect on weight loss was also observed when combined with other medications, such as metformin, sulfonylureas, pioglitazone, and insulin. Weight loss plays a crucial role in the overall health and well-being of patients with T2D, as studies show that weight loss reduces cardiovascular disease risks, such as hyperglycemia, hypertension, and markers of inflammation in patients with T2D [22]. 

While the cardiorenal benefits of using SGLT-2i on patients with T2D are still being examined, studies have determined various benefits offered by specific SGLT-2i. For example, research has shown that administrating patients with empagliflozin for four weeks initially reduced their estimated glomerular filtration rate (eGFR) and then stabilized it [23]. When used in patients with T2D, ertugliflozin was found to reduce the risk of hospitalization due to heart failure and total cardiovascular-related deaths [24]. Additionally, certain SGLT-2i such as canagliflozin and sotagliflozin were observed to cause an overall reduced risk of stroke in T2D patients with CKD [25]. This leads to an overall reduced hospitalization rate due to heart failure and decreased risk of MACE. Canagliflozin has also been proven to lower systolic blood pressure in patients and lead to an overall improvement in blood pressure control, thereby supporting its use in patients with T2D and CKD for end-organ protection and blood pressure-lowering therapy [26]. 

One of the most crucial roles of SGLT-2i involves the reduction of intraglomerular pressure. Studies have shown that taking both SGLT-2i with angiotensin receptor blockers (ARBs) may result in reno-protective benefits despite the difference in their mechanisms [27]. While SGLT-2i result in the vasoconstriction of the afferent arterioles, ARBs cause efferent arterioles to vasodilate. These two mechanisms have a synergistic effect which can lead to a decrease in intraglomerular pressure and hyperfiltration. Ultimately, administering SGLT-2i alongside ARBs will slow the initial decline in the eGFR, contributing to the additive favorable effects on renal outcomes observed in patients with concomitant ARBs. Studies have also shown that SGLT-2i help preserve eGFR in the long term, which can improve cortical oxygenation and preserve tubular mitochondrial function and autophagy [28]. Moreover, early reduction of eGFR by SGLT-2 inhibitors and the pre-use of antidiabetic and antihypertensive may result in improved renal outcomes in the long term [29]. 

Studies have shown that SGLT-2i have been approved for heart failure patients with reduced ejection fraction (HFrEF) even if they do not have diabetes [30]. Perhaps one of the most important benefits of using SGLT-2i is its cardiac benefits, including the reduction of major cardiovascular events, hospitalization rates due to heart failure, and death. These benefits are due to SGLT-2i reducing sodium levels, which in turn decreases plasma volume and blood pressure. This reduction in circulation decreases both preload and afterload, and thus improves cardiac blood flow [31].

The cardiovascular benefits provided by SGLT-2i also extend to improving mitochondrial dysfunction and reducing the production of mitochondrial reactive oxygen species [32]. In addition, SGLT-2i act as a blocker for sodium-hydrogen exchanger in the heart, increasing mitochondrial calcium and decreasing cytoplasmic sodium and calcium levels. In this way, the antioxidant capacity of the mitochondria is improved, and patients will be at a reduced risk of developing cardiac fibrosis and systolic dysfunction [33]. 

Other trials comparing the use of dipeptidyl peptidase 4 (DPP-4) inhibitors to placebo [3-6] showed similar results in cardiovascular conditions. Furthermore, DPP-4 inhibitors resulted in an increased risk of hospitalization due to heart failure. This was particularly observed in the Saxagliptin Assessment of Vascular Outcomes Recorded in Patients with Diabetes Mellitus (SAVOR)-Thrombolysis in Myocardial Infarction (TIMI) 53 trial [5]. The use of luseogliflozin has shown effective results in controlling hyperglycemia in the Dahl-STZ model of diabetic nephropathy. Even though the selective SGLT-2 inhibitor luseogliflozin did not slow down the development of proteinuria, it plays a crucial role in reducing renal hyperfiltration, and glomerular and tubular injury. On the other hand, given that lisinopril reduces blood pressure in this model, using it helped reduce the development of proteinuria, renal hyperfiltration, glomerular injury, and renal fibrosis. When both luseogliflozin and lisinopril were used in combination, effective reductions were observed; the development of proteinuria was reduced much more effectively when both were used in combination, rather than either one alone. 

**Table 2:** Comparisons among specific SGLT-2 inhibitors SGLT2, sodium-glucose cotransporter 2; MACE, major adverse cardiovascular events; eGFR, estimated glomerular filtration rate; UACR, urine albumin to creatinine ratio

SGLT-2 inhibitors	Weight Loss	Blood Pressure	Renal Effect	Cardiac Effect	Stroke Protection
Empagliflozin (EMPA-REG1) [11,15,21,22]	Significantly reduces weight	Significantly reduces hypertension	Helps reduce kidney function progression	-Reduces hospitalization rates due to heart failure and MACE -Helps reduce cardiovascular death	No significant impact
Dapagliflozin (DECLARE-TI MI) [11,15,25]	Significantly reduces weight	Helps reduce systolic blood pressure	Helps reduce kidney function progression	-Helps reduce the risk of cardiovascular death -Helps prevent heart failure progression	No significant impact
Canagliflozin (CANVAS) [11,15,25,26]	Helps reduce weight	Helps improve blood pressure control	Has renoprotective benefits	Helps reduce hospitalization due to heart failure and MACE	Helps reduce the risk of stroke
Ertugliflozin (VERTIS) [11,15,23-25]	Helps reduce weight	Helps reduce systolic blood pressure	-Helps reduce eGFR and UACR -Helps reduce chronic renal replacement therapy and renal death	Helps reduce Heart failure hospitalization and death	No significant impact

## Conclusions

The use of both GLP-1RA and SGLT-2i has been shown to play an instrumental role in decreasing the occurrence of cardiorenal risks in T2D patients. Certain GLP-1RAs such as lixisenatide, liraglutide, semaglutide, exenatide, and albiglutide have been shown to reduce albuminuria and the progression of CKD. In addition, studies have also shown that different GLP-1RAs and SGLT-2i help prevent various cardiovascular diseases. Albiglutide, dulaglutide, and subcutaneous semaglutide have demonstrated a significant reduction in the occurrence of MACE, whereas oral semaglutide, exenatide, and lixisenatide did not. Similarly, studies involving SGLT-2i showed that canagliflozin and empagliflozin reduced MACE and heart failure hospitalization, while dapagliflozin did not. Weight loss is a notable benefit of certain medications within these two classes of drugs. Semaglutide and liraglutide are both FDA-approved for weight loss in patients without diabetes. Lixisenatide and exenatide have shown moderate reductions in weight in several trials, however, they are not FDA approved for weight loss in patients without diabetes. Likewise, empagliflozin and dapagliflozin stimulate significant reductions in weight, while also lowering blood pressure. One of the primary advantages of SGLT-2i are their cardiac benefits, as they are commonly used in the treatment of patients with HFrEF. The mechanism behind this medication class helping decrease mortality rates in heart failure patients involves the reduction in both preload and afterload. The reduction in preload is a result of SGLT-2i promoting diuresis and natriuresis, while a reduction in afterload stems from their vascular changes causing vasodilation and enhanced endothelial function. 

The number of studies conducted on both of these classes of medications highlights the fact that they can have significant effective potential when treating T2D patients who are at risk of cardiorenal complications. Some experts are optimistic about the possibility of these medications being used against diabetic kidney disease. As per the American Diabetes Association, both therapies are ultimately considered the primary choice when treating patients at an increased risk of developing cardiovascular diseases, or those who already suffer from them. Although both glucose-lowering therapies have shown tremendous results that can treat T2D patients at risk of developing cardiorenal complications, further research needs to be conducted on these medications to understand their beneficial properties fully.
